# Clinical characteristics of children and guardians possessing CBP-positive *Streptococcus mutans* strains: a cross-sectional study

**DOI:** 10.1038/s41598-022-22378-8

**Published:** 2022-10-20

**Authors:** Masatoshi Otsugu, Yusuke Mikasa, Takahiro Kitamura, Yuto Suehiro, Saaya Matayoshi, Ryota Nomura, Kazuhiko Nakano

**Affiliations:** 1 Department of Pediatric Dentistry, Osaka University Graduate School of Dentistry, 1-8 Yamada-Oka, Suita, Osaka, 565-0871 Japan; 2 Department of Pediatric Dentistry, Hiroshima University Graduate School of Biomedical and Health Sciences, Hiroshima, Japan

**Keywords:** Molecular biology, Microbiology, Bacteriology, Clinical microbiology

## Abstract

*Streptococcus mutans* is a major etiological agent for dental caries. We previously demonstrated that *S. mutans* strains expressing collagen-binding proteins (CBPs) were related to the pathogenesis of systemic diseases. However, their acquisition and colonization remain unknown. Here, we investigated the detection rates of CBP-positive *S. mutans* strains in children and their guardians to clarify the background for the acquisition and colonization in children. Saliva samples were collected from children and their mothers, and detection of *S. mutans* and collagen-binding genes (*cnm*, *cbm*) was performed by PCR after DNA extraction. The oral status of each child was examined, and their mothers were asked to complete a questionnaire. The isolation rate of Cnm-positive *S. mutans* was significantly higher in mothers than in children. Notably, the possession rates of CBP-positive strains in children were significantly higher in children whose mothers had CBP-positive strains than in children whose mothers did not have these strains. Furthermore, children with CBP-positive strains had a significantly shorter breastfeeding period than children without these strains. The present results suggest that nutritional feeding habits in infancy are one of the factors involved in the acquisition and colonization of CBP-positive *S. mutans* strains.

## Introduction

Dental caries is one of the most common infectious diseases in the world^1^. During the last four decades, its prevalence has decreased, mainly in high-income countries, and particularly among 12-year-old children^2^. Nevertheless, the global prevalence of untreated caries in primary teeth remains at a certain high level^3^. Therefore, further investigations of causes and policies for prevention of dental caries are necessary, especially for early childhood caries.

*Streptococcus mutans* is a principal etiological agent for dental caries^4^. In the oral cavity, these bacteria produce acid and synthesize water-insoluble glucan in the presence of sucrose, facilitating their firm adhesion to tooth surfaces^5^. Adherent *S. mutans* bacteria form biofilms on tooth surfaces that contribute to the development of dental caries^6^. A previous longitudinal study found that earlier acquisition and colonization of *S. mutans* were associated with greater incidence of caries at later ages^7^. *S. mutans* is mainly transmitted vertically from mothers to their children through saliva^8^. The acquisition and colonization of *S. mutans* are considered to occur during early childhood following eruption of the primary teeth, typically by 2–3 years of age in the so-called “window of infectivity”^9^. Meanwhile, *S. mutans* was also detected in the oral cavity of infants aged 6–12 months^10–12^, and particularly in predentate infants^13,14^. Although it is possible that the periods for acquisition and colonization of *S. mutans* differ by genotype and serotype, the details have not been clarified.

Two 120-kDa cell-surface collagen-binding proteins (CBPs; Cnm and Cbm) are related to *S. mutans* collagen-binding activity and involved in its adhesion to dentine, which is linked to an increased risk of dental caries development^15^. CBPs are detected in approximately 10%–20% of *S. mutans* isolates from the oral cavity, and Cnm is more frequently found than Cbm (Cnm: approximately 15% vs. Cbm: approximately 3%)^16–18^. Although the collagen-binding domain of Cnm shows high homology to that of Cbm, the collagen-binding activities of Cbm-positive strains are significantly higher than those of Cnm-positive strains^16^. These two proteins can bind to the extracellular matrix and are involved in the pathogenesis of various systemic diseases, including infective endocarditis, cerebral hemorrhage, IgA nephropathy, and non-alcoholic steatohepatitis^15,19–23^. However, the acquisition and colonization of CBP-positive *S. mutans* strains remain unknown.

Prolonged breastfeeding is thought to increase the risk of dental caries^24^. Furthermore, intake of a sugar-rich diet and the type and virulence of biofilm-forming bacteria could plausibly account for the cariogenicity of long-duration breastfeeding^25^. Human breast milk contains immunological agents such as secretory IgA and IgG, and has anti-inflammatory properties that offer protection to the immature immune systems of both term and preterm infants^26^. Regarding the effect on oral bacteria, human milk was shown to inhibit adhesion of *S. mutans* to saliva-coated hydroxyapatite *in vitro*^27^, with casein, lactoferrin, and secretory IgA in human milk identified as inhibitors^28,29^. Recently, it was reported that human milk oligosaccharides can inhibit the adhesion of certain *S. mutans* strains^30^. To date, the acquisition and colonization of CBP-positive *S. mutans* have not been investigated with a focus on nutritional feeding in infancy.

In the present study, we investigated the detection rates of CBP-positive *S. mutans* strains in children and their guardians to clarify the background for the acquisition and colonization in children, focusing on the influence of nutritional feeding in infancy.

## Results

### Mother–child pairs, clinical characteristics, and caries statuses

Among 316 Japanese mother–child pairs, *S. mutans* was detected in the salivary samples from 100 pairs by PCR after visual evaluation of the plates (31.6%). In total, 1000 clinical strains of *S. mutans* were isolated from the saliva samples of the 100 pairs (5 strains per subject), and the 100 pairs were analyzed for their clinical characteristics, caries statuses, and cariogenic bacterial statuses.

Table 1 shows the clinical characteristics and caries statuses in the 100 mother–child pairs. The mean age of the children was 72.2 ± 25.9 months, the median age was 65.5 (54–83.5) months, and the total percentage of boys was 52.0%. Regarding gestational age, 90.0% of the children were full-term infants, 8.0% were preterm, and 2.0% were post-term. For feeding, 42.0% of children were breastfed exclusively, 4.0% received formula feeding exclusively, and 54.0% received mixed feeding. The median duration of breastfeeding was 12 (5–18) months, and the median duration of formula feeding was 7.5 (0–12) months. Overall, 4.0% of children were never breastfed, 58.0% received formula, and 19.0% were breastfed for ≥ 24 months. The median decayed, missing, and filled teeth (DMFT/dmft) index was 7 (4–11). The mean age of the mothers was 38.4 ± 5.5 years, and the median age was 38.5 (34–42) years. Among the mothers, 17.0% had ≤ 4 caries experiences, 47% had 5–9, and 36.0% had ≥ 10.

**Table 1:** Clinical characteristics and caries statuses in the 100 mother–child pairs.

	Children		Mothers
	(*n* = 100)		(*n* = 100)
Age (months)		Age (years)	
Mean ± SD	72.2 ± 25.9	Mean ± SD	38.4 ± 5.5
Median (IQR)	65.5 (54–83.5)	Median (IQR)	38.5 (34–42)
Sex		Caries experiences	
Male	52 (52.0%)	≤ 4	17 (17.0%)
Female	48 (48.0%)	5–9	47 (47.0%)
Gestational age (weeks)		≥ 10	36 (36.0%)
< 37	8 (8.0%)		
37–42	90 (90.0%)		
> 42	2 (2.0%)		
Exclusive breastfeeding	42(42.0%)		
Exclusive formula feeding	4 (4.0%)		
Mixed feeding	54 (54.0%)		
Duration of breastfeeding (months)			
Median (IQR)	12 (5–18)		
Duration of formula feeding (months)			
Median (IQR)	7.5 (0–12)		
Last feeding age (months)			
Median (IQR)	18 (12–24)		
Prolonged breastfeeding			
≥ 24 months	19 (19.0%)		
DMFT/dmft index			
Median (IQR)	7 (4–11)		

### Comparison of *S. mutans* detection statuses between the mothers and their children

Table 2 shows the numbers of *S. mutans*-like colonies in the saliva samples (MS scores) and the possession rates of CBP-positive strains in the 100 mother–child pairs. No significant differences were found in the MS score between the two groups. The possession rate of CBP-positive strains was higher in mothers (29%) than in children (17%), especially for Cnm (mothers: 27% vs. children: 15%), although the possession rate of CBP-positive strains in all 316 pairs recruited for the study was lower in both mothers and children (mothers: 9.2% vs. children: 5.4%). The possession rate of Cbm-positive strains was the same for mothers and children (2%), and significantly lower than the possession rates of Cnm-positive strains in both mothers (*P* < 0.001) and children (*P* < 0.01). Table 3 shows the numbers of CBP-positive strain in 500 isolates from the 100 mother–child pairs. The isolation rate of CBP-positive strains was higher in mothers (18.6%) than in children (14.0%). Moreover, the isolation rate of Cnm-positive strains was significantly higher in mothers (16.8%) than in children (12.2%) (*P* < 0.05). Meanwhile, the isolation rate of Cbm-positive strains was the same for mothers and children (1.8%), and significantly lower than the isolation rates of Cnm-positive strains in both mothers and children (*P* < 0.001). Table 4 shows the associations between the MS score in mothers and the caries risk in their children (MS score and DMFT/dmft index). The MS score in mothers was related to both the MS score (*P* < 0.05) and the DMFT/dmft index (*P* < 0.05) in their children.

**Table 2:** Numbers of *S. mutans*-like colonies in saliva samples and possession rates of CBP-positive strains in the 100 mother–child pairs.

	Children	Mothers	*P*
	(*n* = 100)	(*n* = 100)	
**Number of *S. mutans*-like colonies in saliva samples (CFU/ml)**			
< 10^4^	37 (37.0%)	40 (40.0%)	
10^4^–10^5^	40 (40.0%)	37 (37.0%)	
10^5^–10^6^	21 (21.0%)	22 (22.0%)	
> 10^6^	2 (2.0%)	1 (1.0%)	0.925
**Number of subjects with CBP-positive strains**	17 (17.0%)	29 (29.0%)	0.064
Cnm +	15 (15.0%)	27 (27.0%)	0.055
Cbm +	2 (2.0%)	2 (2.0%)	1
*P*	0.002	< 0.001	

Fisher’s exact test was used for the statistical analyses.

**Table 3:** Numbers of CBP-positive strains in 500 isolates from the 100 mother–child pairs.

	Children	Mothers	*P*
	(*n* = 500)	(*n* = 500)	
**Number of CBP-positive strain isolates**	70 (14.0%)	93 (18.6%)	0.059
Cnm +	61 (12.2%)	84 (16.8%)	0.048
Cbm +	9 (1.8%)	9 (1.8%)	1
*P*	< 0.001	< 0.001	

Fisher’s exact test was used for the statistical analyses.

**Table 4:** Numbers of *S. mutans*-like colonies in mothers’ saliva samples and their children’s oral statuses (numbers of *S. mutans*-like colonies in saliva samples and DMFT/dmft indexes).

Mothers				
Number of *S. mutans*-like colonies in saliva samples (CFU/ml)		< 10^5^	≥ 10^5^	*P*
		(*n* = 77)	(*n* = 23)	
**Children**				
**Number of *S. mutans*-like colonies in saliva samples (CFU/ml)**				
	< 10^5^ (*n* = 77)	63 (81.8%)	14 (60.9%)	
	≥ 10^5^ (*n* = 23)	14 (18.2%)	9 (39.1%)	0.049
**DMFT/dmft index**				
	< 4 (*n* = 22)	21 (27.3%)	1 (4.3%)	
	4–11 (*n* = 52)	39 (50.6%)	13 (56.5%)	
	> 11 (*n* = 26)	17 (22.1%)	9 (39.1%)	0.030

The percentages in parentheses show the corresponding ratios in the children within each group of mothers.

Fisher’s exact test was used for the statistical analyses.

### Clinical characteristics and caries statuses of the mothers with CBP-positive strains

**Figure 1:**
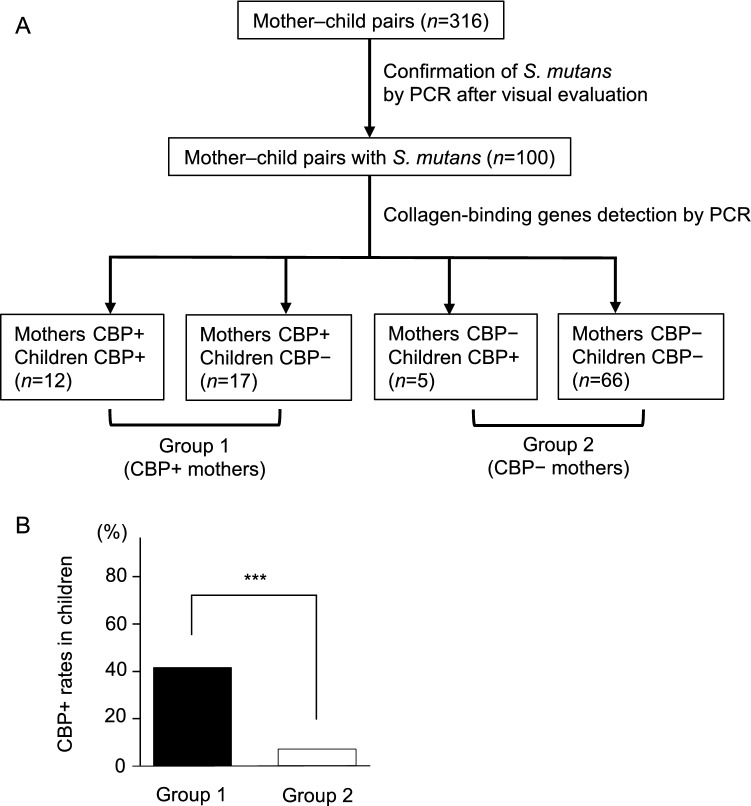
Detection of CBP-positive *S. mutans* in saliva samples from the mother–child pairs in the present study. CBP+ : CBPs-positive *S. mutans* carrier; CBP− : CBPs-positive *S. mutans* non-carrier. (**A**) Details of the CBPs-positive *S. mutans* carriers in the mother–child pairs. (**B**) Possession rates of CBP-positive *S. mutans* in children with CBP-positive *S. mutans* carrier mothers versus children with non-carrier mothers. ***P < 0.001 by Fisher’s exact test.

Table 5 shows the clinical characteristics and caries statuses in the mothers with and without CBP-positive strains. No significant differences were found for age, caries experiences, and MS score between the two groups. However, the possession rate of CBP-positive strains in children was significantly higher in children whose mothers had CBP-positive strains (41.4%) than in children whose mothers did not have these strains (7.0%) (*P* < 0.001) (Fig. 1).

**Table 5:** Clinical characteristics and caries statuses in the mothers with and without CBP-positive strains.

	Mothers		*P*
	CBPs+	CBPs−	
	(*n* = 29)	(*n* = 71)	
**Age (months)**			
Median (IQR)	38 (34 − 43)	39 (35.5 − 42)	0.961^a^
**Caries experiences**			
≤ 4	4 (13.8%)	13 (18.3%)	
5–9	14 (48.3%)	33 (46.5%)	
≥ 10	11 (37.9%)	25 (35.2%)	0.915^b^
**Number of *S. mutans*-like colonies in saliva samples (CFU/ml)**			
< 10^5^	20 (69.0%)	57 (80.3%)	
≥ 10^5^	9 (31.0%)	14 (19.7%)	0.338^c^

^a^The Mann–Whitney U test was used for the statistical analysis.

^b^Fisher’s exact test was used for the statistical analysis.

^c^The chi-square test was used for the statistical analysis.

### Clinical characteristics, caries statuses, and infant feeding habits in children with CBP-positive strains

Table 6 shows the clinical characteristics, caries statuses, and infant feeding habits in the children with and without CBP-positive strains. No significant differences were found in age, sex, gestational age, DMFT/dmft index, and MS score between the two groups. However, children possessing CBP-positive strains had significantly shorter breastfeeding periods than children without these strains (*P* < 0.05). Furthermore, the rate of exclusive formula feeding during infancy was significantly higher in children possessing CBP-positive strains than in children without these strains (*P* < 0.05). No significant differences were found between the two groups for any other items related to nutritional history during infancy.

**Table 6:** Clinical characteristics, caries statuses, and infant feeding habits in the children with and without CBP-positive strains.

	Children		*P*
	CBPs +	CBPs −	
	(*n* = 17)	(*n* = 83)	
**Age (months)**			
Median (IQR)	73 (59 − 90)	65 (54 − 81.5)	0.471^a^
**Sex**			
Male	9 (52.9%)	43 (51.8%)	
Female	8 (47.1%)	40 (48.2%)	1^b^
**Gestational age (weeks)**			
< 37	0 (0.0%)	8 (9.6%)	
37–42	17 (100.0%)	73 (88.0%)	
> 42	0 (0.0%)	2 (2.4%)	0.549^b^
**DMFT/dmft index**			
Median (IQR)	7 (4–12)	7 (4–10)	0.675^a^
**Number of** ***S. mutans*-like colonies in saliva samples (CFU/ml)**			
< 10^5^	13 (76.5%)	64 (77.1%)	
≥ 10^5^	4 (23.5%)	19 (22.9%)	1^b^
**Exclusive breastfeeding**	4 (23.5%)	38 (45.8%)	0.155^c^
**Exclusive formula feeding**	3 (17.6%)	1 (1.2%)	0.015^b^
**Mixed feeding**	10 (58.8%)	44 (53.0%)	0.864^c^
**Duration of breastfeeding (months)**			
Median (IQR)	6 (1–13)	12 (6–19)	0.027^a^
**Duration of formula feeding (months)**			
Median (IQR)	12 (6–15)	3 (0–12)	0.113^a^
**Last feeding age (months)**			
Median (IQR)	16 (12–18)	18 (12–24)	0.476^a^
**Prolonged breastfeeding**			
≥ 24 months	1 (5.9%)	18 (21.7%)	0.183^b^

^a^The Mann–Whitney U test was used for the statistical analyses.

^b^Fisher’s exact test was used for the statistical analyses.

^c^The chi-square test was used for the statistical analyses.

## Discussion

*S. mutans* is a major causative pathogen for dental caries, and its acquisition and colonization occur at a very early stage in life, mainly through vertical transmission from mothers to their infants^9^. Earlier acquisition and colonization of *S. mutans* is associated with greater incidence of caries at later ages^7^. Therefore, prevention of *S. mutans* acquisition and colonization at an early stage is important for long-term health of the oral cavity. Furthermore, CBP-positive *S. mutans* strains are thought to be associated with various systemic diseases, including infective endocarditis, cerebral hemorrhage, IgA nephropathy, and non-alcoholic steatohepatitis^19–23^. However, no preventive strategies for these systemic diseases have been established. For infective endocarditis, no clear evidence has been obtained to date for the effectiveness of prophylactic antibiotic administration^31,32^. Meanwhile, our recent study showed that CBP-positive *S. mutans* killed by amoxicillin retained a certain level of pathogenicity^33^. Therefore, the fundamental preventive strategy for these systemic diseases is to prevent the colonization of CBP-positive *S. mutans* in the oral cavity. In the present study, we evaluated CBP-positive *S. mutans* strains isolated from mother–child pairs to clarify the background for the acquisition and colonization in children.

There were no significant differences in the MS score between the mothers and their children, consistent with the findings in a previous study^34^. However, in the evaluation of caries risk between the mothers and their children, the MS score in mothers was related to both the MS score (*P* < 0.05) and the DMFT/dmft index (*P* < 0.05) in their children. It has been proposed that there is a quantitative correlation between the MS scores in mothers and their children, and that a high MS score in mothers is a strong risk indicator for caries in their children^35,36^. Family-level factors, including transmission of cariogenic bacteria, and eating habits, such as sugar consumption, may have resulted in the high DMFT/dmft index values in children in the present study.

The possession rate of CBP-positive strains was higher in mothers (29%) than in children (17%), especially for Cnm (mothers: 27% vs. children: 15%). The possession rate of Cbm-positive strains was the same for mothers and children (2%), and significantly lower than the possession rates of Cnm-positive strains in both mothers (*P* < 0.001) and children (*P* < 0.01). In previous studies, the possession rates of Cnm-positive strains were reported to be approximately 20% in general^18^ and 7.1%–20% in children^17,37–40^. The possession rates of Cbm-positive strains in the present study were consistent with those in previous studies^17,37,39^. However, the possession rates of CBP-positive strains in all 316 pairs recruited for the study were lower (mothers: 9.2% vs. children: 5.4%) than those in previous studies^17,37–40^.

In the present study, the isolation rate of CBP-positive strains was higher in mothers (18.6%) than in children (14.0%). Furthermore, the isolation rate of Cnm-positive strains was significantly higher in mothers (16.8%) than in children (12.2%) (*P* < 0.05). Nomura et al.^41^ reported that the rates in adults were higher than those in children. However, the present study is the first to demonstrate that children had a significantly lower detection rate than their mothers. These findings may indicate that the *cnm* gene is acquired with age by horizontal gene transfer or that Cnm-positive strains tend to be transmitted horizontally. In contrast, the isolation rate of Cbm-positive strains was the same for mothers and children (1.8%), and significantly lower than the isolation rates of Cnm-positive strains in both mothers and children (*P* < 0.001). In previous studies, *cnm* was detected in approximately 10%–20% of all isolates^16,18,20,38,42–45^, and *cbm* was more rarely detected in < 3% of isolates^16^. The results of the present study were consistent with these previous studies.

No significant differences were found in caries experiences and MS score between the mothers with and without CBP-positive strains. Therefore, CBPs may not be an important factor for the development of dental caries. However, the possession rate of CBP-positive strains in children was significantly higher in children whose mothers had CBP-positive strains than in children whose mothers did not have these strains (*P* < 0.001). This tendency was consistent with that in a previous study, in which only Cnm-positive strains were investigated and the sample size was smaller than that in the present study^41^. The findings indicate that vertical transmission of CBP-positive strains may occur in mother–child pairs.

In the present study, we evaluated the clinical characteristics of children possessing CBP-positive *S. mutans* strains. No significant differences were found in age, sex, gestational age, DMFT/dmft index, and MS score between children with and without CBP-positive *S. mutans* strains. However, children possessing CBP-positive strains had a significantly shorter breastfeeding period than children without these strains (*P* < 0.05), and the rate of exclusive formula feeding during infancy was significantly higher in children possessing CBP-positive strains than in children without these strains (*P* < 0.05). The acquisition and colonization of *S. mutans* have been considered to occur at approximately 19–31 months after birth^9^, although some studies detected *S. mutans* in the oral cavity of infants aged < 12 months^10–14^. Furthermore, because CBP-positive strains can adhere to not only dentine but also the extracellular matrix due to their specificity, they have the potential to colonize the oral cavity before the occurrence of tooth eruption^15^. Meanwhile, specific components of human milk, such as secretory IgA, casein, lactoferrin, and oligosaccharides, were shown to inhibit the adhesion of some *S. mutans* strains in vitro, although the data were limited and not necessarily consistent in the previous studies^28–30^. Based on these reports, the present results suggest that certain components of breast milk may be directly or indirectly involved in the colonization of CBP-positive strains and that CBPs may have the ability to adhere to these components. However, the present results could also be interpreted to suggest that acquisition of CBPs was promoted by artificial feeding. Further studies focusing on the effects of human milk on the acquisition and colonization of CBP-positive strains are warranted.

The present study had some limitations. First, although the sample size was relatively large, it remained small for evaluation of the characteristics of CBPs-positive *S. mutans* carriers, due to their low frequency. Consequently, it was difficult to conduct statistical analyses on these characteristics by conventional methods, considering the multiple factors involved. Recently, a loop-mediated isothermal amplification method for rapid detection of Cnm-positive *S. mutans* was reported, and this method may be useful for large-scale surveys^46^. Second, the severity of caries did not necessarily coincide with the DMFT/dmft index in the children, due to the wide age range of the children in the study. In addition, although the questionnaire in the present study was used as a part of daily clinical practice for children, the mothers’ exact caries status should have been clinically evaluated by oral examination, rather than using the questionnaire. Third, molecular analyses such as multi-locus sequence typing, arbitrarily primed PCR, chromosomal DNA fingerprinting, and chromosomal DNA restriction fragment length polymorphism should be applied to accurately prove the transmission of *S. mutans*^47^. Fourth, there were limitations in evaluating vertical transmission, because information on the primary caregivers was not investigated in the present study. Finally, not all CBP-positive *S. mutans* necessarily express CBPs, which may have biased the results of the study. Nevertheless, the only strategy for the prevention of systemic diseases associated with CBP-positive *S. mutans* is to stop the colonization of CBP-positive *S. mutans* in the oral cavity, and thus it is extremely meaningful for quality of life improvement to clarify the colonization mechanisms of CBP-positive *S. mutans*. Within the limitations of this experimental study, the following conclusions can be drawn: (1) the isolation rate of Cnm-positive *S. mutans* was significantly higher in mothers than in children; (2) CBP-positive *S. mutans* can be vertically transmitted in mother–child pairs; and (3) children possessing CBP-positive *S. mutans* had a significantly shorter breastfeeding period than children without these strains. We consider that nutritional feeding habits in infancy are one of the factors involved in the acquisition and colonization of CBP-positive *S. mutans* strains.

## Materials and methods

### Ethics statement

This study was conducted with full adherence to the Declaration of Helsinki. The study protocol was approved by the Ethics Committee of Osaka University Graduate School of Dentistry (Approval Number H30-E3). All parents were informed of the study contents through the provision of written forms and verbal explanations, and written informed consent for study participation was obtained from all parents. Verbal agreement for participation was obtained from all children, and written informed assent was also obtained from children aged > 6 years.

### Subjects and saliva sample collection

In total, 316 mother–child pairs who attended the Pediatric Dentistry Clinic at Osaka University Dental Hospital, Suita, Osaka, Japan, from 2018 to 2021 were analyzed. The inclusion criteria were that neither the mothers nor their children had systemic diseases and antibiotic administration within the 3 previous months, and that the children were aged 3–11 years. Unstimulated saliva samples were obtained by asking the subjects to expectorate 2–3 ml of saliva into a sterile 50-ml tube between 9 am and 12 noon. The saliva samples were kept on ice for no longer than 3 h prior to bacterial isolation.

### Bacterial isolation

*S. mutans* strains in the saliva samples were isolated and counted as previously described^34^. The saliva samples underwent tenfold serial dilution with saline solution, and were plated on mitis salivarius (MS) agar (Difco Laboratories, Detroit, MI, USA) plates containing bacitracin (0.2 U/ml; Sigma Chemical Co., St. Louis, MO, USA) and 15% (wt/vol) sucrose (MSB agar plates). All plates were incubated at 37 °C for 2 days under an atmosphere of 95% N_2_ and 5% CO_2_. *S. mutans*-like colonies on the MSB agar plates were detected by observation of the rough colony morphology^48^. The number of *S. mutans*-like colonies in the saliva samples was defined as the MS score and expressed as colony-forming units per milliliter (CFU/ml) of saliva. Counts higher than 10^5^ CFU/ml of *S. mutans* in saliva were defined as high levels and high risk for dental caries^49^. For each subject, five rough *S. mutans*-like colonies on MSB agar plates were randomly chosen, inoculated into brain heart infusion broth (BHI; Difco Laboratories), and cultured at 37 °C for 18 h for genomic DNA extraction.

### DNA extraction

Genomic DNA was extracted from each strain as previously described^50^. Briefly, the bacterial cells cultured in BHI were collected and incubated with 62.5 μl of lysozyme chloride from chicken egg white (2.0 mg/ml; Sigma-Aldrich Co., St. Louis, MO, USA) and 0.25 μl of lysozyme hydrochloride from chicken egg white (10 mg/ml; Fujifilm Wako Pure Chemical Industries, Osaka, Japan) for 90 min at 37 °C. For genomic DNA extraction, the samples were incubated in 600 μl of Cell Lysis Solution (Qiagen, Düsseldorf, Germany) at 80 °C for 5 min, followed by addition of 3 μl of RNase A (10 mg/ml; Qiagen) and incubation at 37 °C for 30 min. Next, 200 μl of Protein Precipitation Solution (Qiagen) was added and vortexed vigorously for 20 min, followed by centrifugation at 10,000 × *g* for 3 min. The supernatant was combined with 600 μl of isopropanol (Fujifilm Wako Pure Chemical Industries) and centrifuged. The precipitate was resuspended in 70% ethanol (Fujifilm Wako Pure Chemical Industries), centrifuged, combined with 100 μl of DNA Hydration Solution (Qiagen), and stored as a DNA extract.

### Confirmation

Confirmation of *S. mutans* and detection of collagen-binding genes (*cnm* and *cbm*) were carried out by PCR using TaKaRa Ex Taq polymerase (TAKARA BIO, Shiga, Japan), with *S. mutans-*specific primers (forward, 5′-GGC ACC ACA ACA TTG GGA AGC TCA GTT-3′; reverse, 5′-GGA ATG GCC GCT AAG TCA ACA GGA T-3′)^51^, *cnm-*specific primers (forward, 5′-GAC AAA GAA ATG AAA GAT GT-3′; reverse, 5′-GCA AAG ACT CTT GTC CCT GC-3′)^41^, or *cbm-*specific primers (forward, 5′-GAC AAA CTA ATG AAA TCT AA-3′; reverse, 5′-GCA AAA ACT GTT GTC CCT GC-3′)^16^, template DNA, and 1.5 mM MgCl_2_, according to the supplier’s protocols. Amplification was performed using the following parameters: *S. mutans*, 30 cycles of denaturation at 98 °C for 10 s, and primer annealing and extension at 70 °C for 1 min; *cnm* and *cbm*, initial denaturation at 95 °C for 4 min, followed by 30 cycles of 95 °C for 30 s, 60 °C for 30 s, and 72 °C for 2 min, and a final extension at 72 °C for 7 min. The PCR products were subjected to electrophoresis in 1.5% or 0.7% agarose gels in Tris–acetate-EDTA buffer. The gels were stained with 0.5 μg/ml ethidium bromide and photographed under ultraviolet illumination. MT8148 (*cnm*-negative, *cbm*-negative), TW295 (*cnm*-positive) and SA31 (*cbm*-positive) were used as controls in the PCR reactions. Samples with positive reactions for *cbm* or *cnm* were defined as Cbm-positive or Cnm-positive, respectively.

### Clinical examination and questionnaire for mothers

The oral status of each child was examined using a mirror and an explorer under a dental operation light. For each child, the DMFT/dmft index was calculated according to the criteria established by the World Health Organization^52^. Because some children were in the mixed dentition phase, it was decided to evaluate dmft for primary teeth and DMFT for permanent teeth in total. In addition, all mothers were asked to complete a questionnaire about their age and caries experiences, as well as the child’s gestational age and nutritional history in infancy (durations of breastfeeding and/or formula feeding). Prolonged breastfeeding was defined as breastfeeding for ≥ 24 months, based on the risk of having dental caries^24^. The questionnaires were collected after confirming that all items were complete.

### Statistical analysis

The sample size was calculated using the G* Power program, version 3.1.9.6^53^. A value of 0.3 (Cohen’s *w*) was used for the sample size calculation to achieve > 80% power with an anticipated correlation of > 0.2 and a significance level of 5%^54^. Overall, 61 or more *S. mutans* carrier pairs were required to compare the detection rates of CBP-positive *S. mutans* between mothers and children, and 88 or more *S. mutans* carrier children were required to clarify the background for the acquisition and colonization. The possession rates of *S. mutans* in all mother–child pairs were calculated, followed by the detection rates of CBP-positive strains in the *S. mutans* carrier pairs. Intergroup differences in the bacterial characteristics in saliva samples were analyzed using the chi-square test or Fisher’s exact test where appropriate. Nutritional history in infancy was compared using the Mann–Whitney U test and chi-square test. All data were analyzed using R version 4.1.1^®^ (R Foundation for Statistical Computing Vienna, Austria; https://www.R-project.org/), and the level of statistical significance was set at *P* < 0.05.

## Data Availability

All data generated or analyzed during this study are included in this published article.
